# Histological and Immunohistochemical Evaluation of the Efficacy of a New Cosmetic Formulation in the Treatment of Skin Photoaging

**DOI:** 10.1155/2017/8407247

**Published:** 2017-01-12

**Authors:** M. T. Truchuelo, N. Jiménez, L. Miguel-Gomez, A. Hermosa, N. Sánchez-Neila, J. Cuevas

**Affiliations:** ^1^Clinica Grupo Pedro Jaén, Madrid, Spain; ^2^Ramón y Cajal University Hospital, Madrid, Spain; ^3^Guadalajara University Hospital, Guadalajara, Spain

## Abstract

*Objective*. Mechanism of action of cosmetic products is not often studied. The aim of this study is to determine the histological, immunohistochemical, and clinical changes of a new cosmetic formulation.* Methods*. Prospective, single-blind, patient-controlled, randomized study in 10 volunteers with mild to moderate skin photoaging on the back of their hands. The product was applied on one hand and a standard cream on the other hand, twice a day for three months. Standardized photographs were taken on basal (T0) and final visit (T1) and skin biopsies were performed. Changes on histological and immunohistochemical markers were studied. Subjective clinical changes were determined.* Results*. After treatment, a 26.3% improvement on epidermal thickness was detected and a significant increase on collagens I and III, elastin, and fibronectin fibers was achieved (*p* < 0.05). As the expression of MMPs remained stable, this improvement of dermal matrix was attributed to the stimulation of their synthesis. A significant clinical improvement on the treated hand was obtained, compared to control hand.* Conclusion*. This new cosmetic product with combination of three registered technologies (IFC-CAF, WGC, and RetinSphere), focused on regenerating dermal matrix and activating proliferation of skin cells, has shown to be efficient in the reversion of skin photoaging.

## 1. Introduction

There are two basic types of skin aging, namely, intrinsic and extrinsic aging. The former, also called chronological aging, is related to telomere shortening and to the accumulation of reactive oxygen species (ROS), mainly generated by mitochondrial metabolism, which the cells are unable to neutralize [1]. Slight rhytides and dermal atrophy with loss of elasticity are clinical signs of this type of skin aging [1]. By contrast, in extrinsic aging or photoaging, the long-term effects of exposure to ultraviolet radiation (UVR) and other environmental agents such as gravity, pollution, diet, tobacco, illness, or stress, favor the development of the clinical signs of extrinsic aging like pigmentation changes, telangiectasias, increment of corneal size, deep wrinkles, actinic keratosis, and precancerous and cancerous skin lesions [2].

Aged skin is microscopically characterized by cellular senescence, which determines a decrease in the proliferation and physiology of keratinocytes and fibroblasts. This results in decreased epidermal renewal and reduced synthesis of dermal extracellular matrix (ECM) components, such as collagen and elastin. Additionally, degeneration of the ECM is accelerated due, in part, to the increased expression of metalloproteinases (MMPs), mainly MMP-1 and MMP-3 [3]. Altogether, these changes end with epidermal and dermal atrophy and, finally, impairment in the structural and physiological properties of the tissue [4, 5].

A new product has recently been designed based on a combination of three technologies, each of which tackles a clinical sign of photoaging, thus trying to provide a global approach to this skin condition: IFC-CAF (Cellular Activating Factor), WGC (Wharton Gel Complex), and RetinSphere.

*Mechanism of Action of the Different Technologies of the Study Product*

*RetinSphere Technology*

Normalization of epidermal proliferation and follicular keratinization
Anti-inflammatory properties
Enhancing penetration of products

*IFC-CAF*

Antioxidant effects
Proliferation and migration of cutaneous stem cells, keratinocyte cells, and fibroblasts.
Stability of DNA

*WGC*

Antioxidant effects
Proliferation and migration of fibroblasts
Synthesis collagens I, III, and VII and elastin.

RetinSphere encompasses the association of two topical retinoids: retinol glycospheres and hydroxypinacolone retinoate. It has anti-inflammatory properties and normalizes epidermal proliferation. In addition, it enhances the penetration of the other components of the cosmetic formulation [6]. This ingredient has demonstrated to increase skin elasticity and brightening and to decrease wrinkles and pigmentation of photoaged skin [7]. Furthermore, other studies have demonstrated that RetinSphere improves uneven complexion and telangiectasias [8].

*IFC-Cellular Activating Factor (IFC-CAF)* is a cosmetic ingredient obtained from a lysate of the eggs of gastropods from the Helicidae family, with powerful regenerative properties, which has demonstrated to slow down stem cell proliferation [9] and to induce skin stem cells migration in a time- and dose-dependent manner improving skin homeostasis. Furthermore, this ingredient promotes a better organization of cytoskeletal proteins (F-actin and vimentin) and promotes the production of ECM components (fibronectin, collagen I, and MMPs) and the adhesion to cell-substrate vinculin protein. The treatment with this factor decreases the expression of the aging-related markers *β*-galactosidase, p53, and p16INK4 in senescent fibroblasts and improves cell survival after UVB irradiation and nuclear repair in HaCaT cells [10]. Additionally, it protects HaCaT cells from genomic damage, as has been demonstrated by the reduction of the levels of the phosphorylated form of histone H2Ax following irradiation with UVB radiation [10].

*Wharton Gel Complex* (WGC) is constituted by a natural mixture of glycosaminoglycans (GAGs) obtained from Wharton's jelly from the umbilical cord of swine. WGC stimulates proliferation of fibroblasts and exhibits a high chemotactic activity, stimulating their migration to the site of injection, as shown through a transwell migration assay.

The analysis of ECM components proved the ability of WGC to increase the levels of collagen I and fibronectin fibers, as well as the synthesis of collagens III and VII and hyaluronic synthases 1 and 3 (HAS1 and HAS3) [11]. Thus WGC is an effective ingredient with regenerative properties.

The main objective of the study was to evaluate the efficacy in improving facial photodamage of a cosmetic product containing, among others, the combination of these three active ingredients, which will be assessed by analyzing the improvement of the expression of several immunohistochemical markers.

The evaluation of clinical changes, such as pigmentation and hydration, after using the product, as well as the degree of improvement perceived by investigator and patient, were deemed as secondary variables. Adverse events were monitored throughout the study.

## 2. Study Population and Methods

This is a prospective, single-blind, randomized study where the volunteers acted as their own comparators self-administering the product only in one hand, while applying their standard hydration regime on the other hand. The patients also acted as their own internal control before and after treatment. Ten volunteers were recruited.

### 2.1. Inclusion Criteria

- Women aged between 45 and 64 years.
- Absence of treatment on their hands in the last 3 months.
- No desire to get pregnant in the forthcoming months and use of contraceptives.
- No concomitant diseases.
- No administration of another topical or systemic product that may interfere with or affect the results.
- No allergy to the product ingredients.

### 2.2. Treatment Regime

The treatment included the application of the new formulation (Endocare Cellage®/CellPro®—IFC SA) twice a day for three months on the dorsal area of the assigned hand for each volunteer. On the other hand, a standard cream was applied.

### 2.3. Clinical Assessment

Clinical assessment of both hands and skin biopsy of the hand that received the study product were performed at basal visit (T0) and following three months of treatment (T1). No biopsies were performed in the untreated hand for ethical reasons.

At both time points, classical clinical photography was carried out with Reveal® System with RBX technology and biopsies were performed. To camouflage postbiopsy wounds at the base of the fifth metatarsal, a bandage was placed covering them, and the same was done in the same place in the contralateral hand so the evaluation remained blinded. Two different dermatologists, who were different from the one who performed the biopsies, performed clinical assessments independently.

The following parameters were recorded:

- Age.
- Phototype.
- Degree of hands photoaging as perceived by the patient (0 to 3) (see “*Clinical Evaluation Graduation Assessed by Patient and Investigators”*). Photoaging signs that the patient should evaluate included rhytides, roughness, lack of brightness, lack of elasticity, lentigines, and desquamative lesions.
- Degree of pigmentation and hydration as perceived by the investigator (0 to 3) (see “*Clinical Evaluation Graduation Assessed by Patient and Investigators”*).
- Degree of improvement of photoaging as perceived by the patient (−2 to 3) (see “*Clinical Evaluation Graduation Assessed by Patient and Investigators”*).
- Degree of improvement of photoaging perceived by the investigator (−2 to 3) (see “*Clinical Evaluation Graduation Assessed by Patient and Investigators”*).

Adverse effects were also reported across the study and scaled (0 to 2): desquamation, itching, burning, erythema, dryness, or others (see “*Clinical Evaluation Graduation Assessed by Patient and Investigators”*).

*Clinical Evaluation Graduation Assessed by Patient and Investigators*

Pigmentation and hydration by investigator were as follows: 0: none, 1: light, 2: moderate, and 3: intense.
*Photoaging* changes described by investigator were as follows: 0: none, 1: slight improvement, 2: moderate improvement, 3: intense improvement, −1: slight worsening, and −2: intense worsening.
Patient and Investigator Global Assessment were as follows: 0: none, 1: slight improvement, 2: moderate improvement, 3: intense improvement, −1: slight worsening, and −2: intense worsening.
Adverse Events: Desquamation, Itching, Burning, Erythema, Dryness, and Others were as follows: 0: none, 1: moderate, and 2: intense.

### 2.4. Histological Assessment

The pathologist who evaluated the biopsies was not informed whether the samples were before or after treatment. Positive and negative controls were included. After paraffin fixation, 4 *μ*M slices were cut for histological and immunohistochemical staining.

Biopsies were stained with hematoxylin-eosin to assess epidermal and dermal thickness and morphology.

Orcein staining was performed to evaluate the expression of elastic fibers before and after treatment. Semiquantitative analysis of elastosis (0–3) and elastic fibers density in reticular dermis were assessed.

Mucopolysaccharides of reticular dermis were semiquantitatively assessed (0–3) by colloidal iron staining.

Immunohistochemical analysis was also performed to address the expression of several markers: CD31 (monoclonal Ab. Clon JC70A, prediluted), Ki67 (monoclonal Ab. Clon MIB1, prediluted), collagen I (polyclonal Ab., diluted 1/100), collagen III (monoclonal Ab. Clon IE7-D7, diluted 1/600), fibronectin (polyclonal Ab, diluted 1/250), metalloproteinase 1 (MMP-1, Monoclonal Ab. Clon EP1247Y, diluted 1/75), and metalloproteinase 3 (monoclonal Ab. Clon 148-1A 3, diluted 1/75).

Cells positive for these markers were quantified and expressed as positive cells/mm2 (40x), and the expression of collagen fibers and fibronectin was semiquantitatively evaluated, scoring the microscopy images from 0 (none) to 3 (high levels).

### 2.5. Statistical Analysis

The comparisons between the effects of the cosmetic product and the pre- and posttreatment effects were evaluated with semiquantitative or quantitative variables, depending on the immunohistochemical biomarker. For each variable in the study, a nonparametric Wilcoxon test (paired samples) was used to contrast the possible significant differences between the period after and that before treatment and between treated and nontreated side. Results are expressed as the mean ± standard deviation of each clinical and histological variable. A *p* value less than 5% (*p* < 0.05) was considered as significant (^*∗*^*p* ≤ 0.05, ^*∗∗*^*p* ≤ 0.01, compared to pretreatment). All the calculations and tests were made by using the software SPSS, V21.0.

## 3. Results

A total of 10 women aged between 45 and 64 years were included. All of them completed the study.

### 3.1. Clinical Assessments

**Figure 1:**
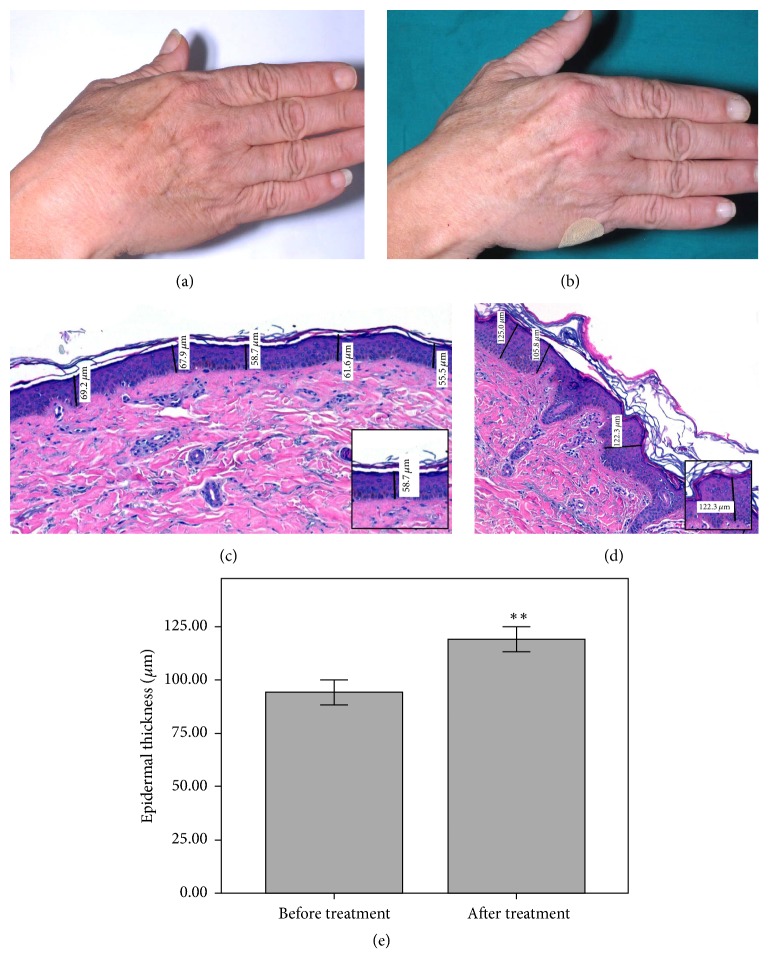
Clinical images before (a) and after treatment (b) showing more brightness and less rhytides after treatment. Hematoxylin-eosin image before (c) and after treatment (d), showing the improvement on epidermal thickness (40x magnification). Epidermal thickness increases by 26.3% after treatment compared to baseline (^*∗∗*^*p* ≤ 0.01) (e).

The clinical and photographic analysis with RBX technology is shown in Figure 1, with an example image of a hand before and after treatment with the study product. The clinical images were analyzed by two blinded independent investigators, who reached an 80% coincidence when identifying the hand that had received the treatment.

Regarding pigmentation, there were no significant differences between both hands at baseline; however, a mild improvement, which showed a trend to significance (*p* = 0.083), was observed in the pigmentation of the treated hand compared to the untreated hand at the end of the treatment (Table 1).

Notably, very significant differences were accomplished in the degree of hydration in the treated hand compared to the untreated hand (3.0 versus 1.7; *p* = 0.006) and compared to baseline (*p* = 0.008), while both hands showed exactly the same hydration at baseline (1.95, i.e., moderate hydration) (Table 1).

### 3.2. Subjective Clinical Improvement

**Figure 2:**
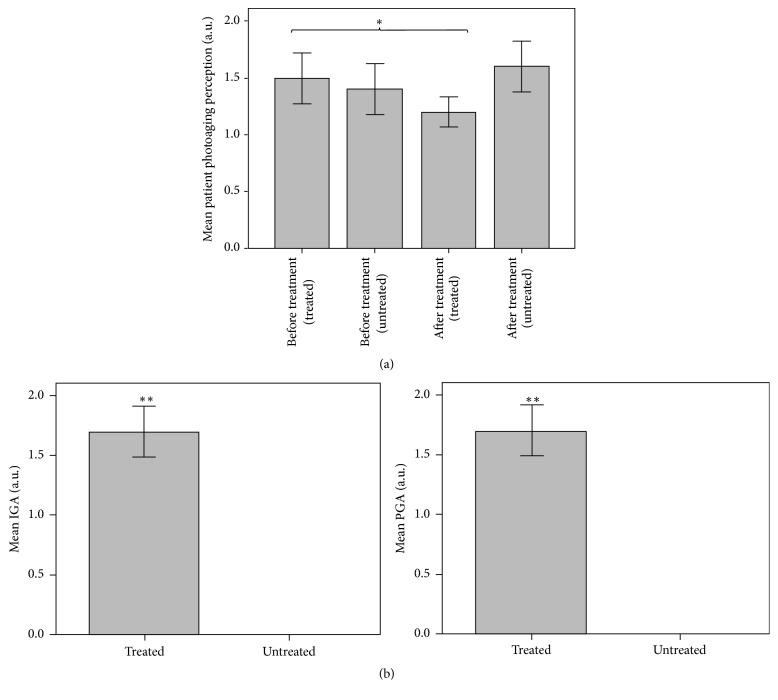
Perception of the degree of photoaging perceived by the patient showing an improvement on the self-perception of photoaging after treatment compared to baseline (^*∗*^*p* ≤ 0.05) (a). Patient and Investigator global assessment (b) demonstrating a significant difference between the improvement experienced by the treated hand compared to the untreated hand (^*∗∗*^*p* ≤ 0.01).

The perception of the degree of aging perceived by the patient was scored similarly in both hands at baseline (average 1.45, i.e., mild and moderate), objectifying a significant improvement in the treated hand compared to untreated hand after treatment (1.2 versus 1.6; *p* = 0.046) (Figure 2(a)).

Both patient and investigator assessment showed significant improvement of the treated hand after completing the treatment with the new product (Figure 2(b)) (*p* = 0.004). The degree of improvement after treatment perceived by both the investigator and the patient resulted in a very significant amelioration of photoaging perception in the treated compared to the untreated hand. This improvement of the treated hand was classified as severe or moderate in 6 patients and mild in 4 of them. By contrast, in the untreated hand, neither the investigator nor the patient observed any improvement.

No adverse effects were reported in any case.

### 3.3. Histological Assessments

The hematoxylin-eosin staining of the biopsies performed in the treated hand showed obvious differences in many of the samples regarding the epidermal and dermal thickness of the treated hand after treatment compared to baseline, although only epidermal thickness increased significantly. At baseline the average epidermal thickness was 94.4 *μ*m, while it was 119.2 microns after treatment, showing a 26.3% statistically significant increase (*p* = 0.008) (Figure 1). However, a slight increase of 8.2% was observed in dermal thicknesses in biopsies before and after treatment (1.10 mm and 1.19 mm, resp.) but it did not reach statistical significance (*p* = 0.093).

**Figure 3:**
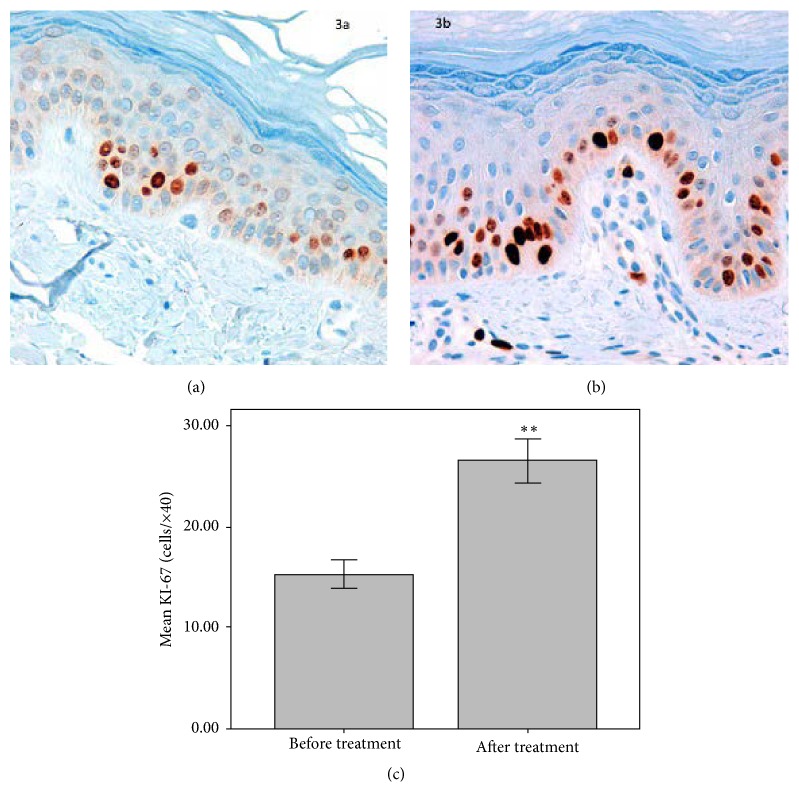
Immunohistochemical staining with Ki67 proliferation marker, before (a) and after (b) treatment showing the improvement of the proliferation index (40x magnification) at the end of the study. Quantification of Ki67 positive cells showed a 73.2% increase after treatment compared to baseline (^*∗∗*^*p* ≤ 0.01) (c).

Interestingly, the expression of the cell proliferation marker Ki67 (cells/×40) after treatment was 73.2% higher compared to baseline (*p* = 0.007) (Figure 3).

**Figure 4:**
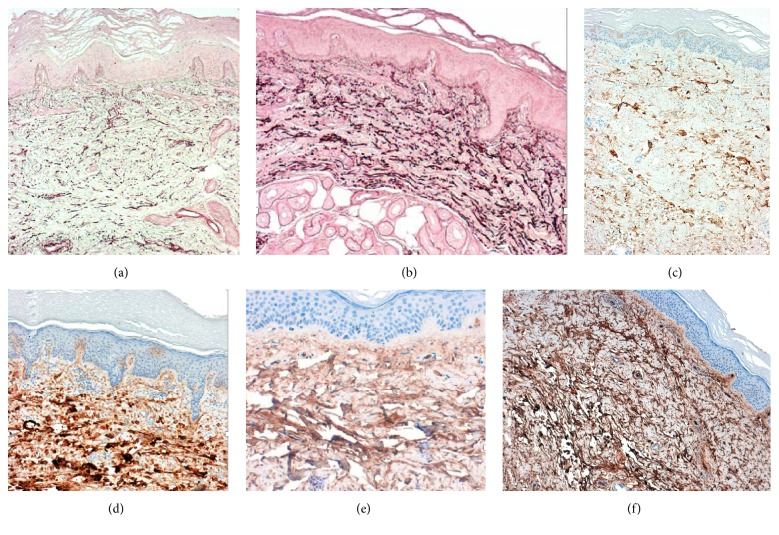
Immunohistochemical staining showing the significant increase of elastic fibers after the cosmetic treatment (b) compared to pretreatment (a). Immunohistochemical staining showing the expression of collagen III before (c) and after treatment showing a significant increase of this ECM marker (d). Immunohistochemical staining for fibronectin showing its expression before treatment (e) and the important increase after the cosmetic treatment (f) (40x magnification).

**Figure 5:**
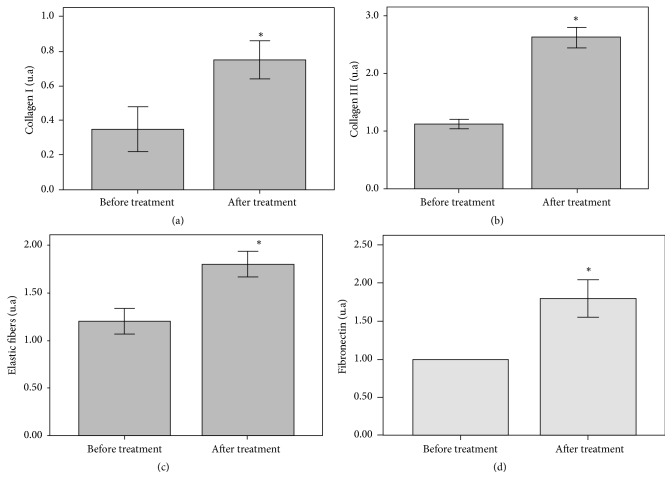
Semiquantitative analysis of immunohistochemistry images of collagens I (a) and III (b), elastin (c), and fibronectin (d) before and after the cosmetic treatment, showing increases of 50.0%, 100.0%, 116.6%, and 80.0%, respectively, in the levels of each ECM component at the end of the treatment compared to baseline (^*∗*^*p* ≤ 0.05).

Even though dermal thickness did not change significantly, an important increase in dermal ECM fibers was observed after treatment compared to baseline. The histological study showed that elastin density increased 50.0% following 3 months of treatment compared to baseline (*p* = 0.014) (Figures 4(a), 4(b), and 5). Likewise, a significant increase was observed in collagen fibers types I (100.0% higher after treatment, *p* = 0.011) and III (116.6% higher after treatment, *p* = 0.011) (Figures 4(c), 4(d), and 5). In a similar way, fibronectin expression showed a significant increase of 80.0% in the treated hand following treatment compared to baseline (*p* = 0.023) (Figures 4(e), 4(f), and 5).

Notably, no significant changes were observed in the expression of metalloproteinases 1 and 3 after treatment. No inflammatory infiltrate was observed in any volunteer before or after treatment.

**Figure 6:**
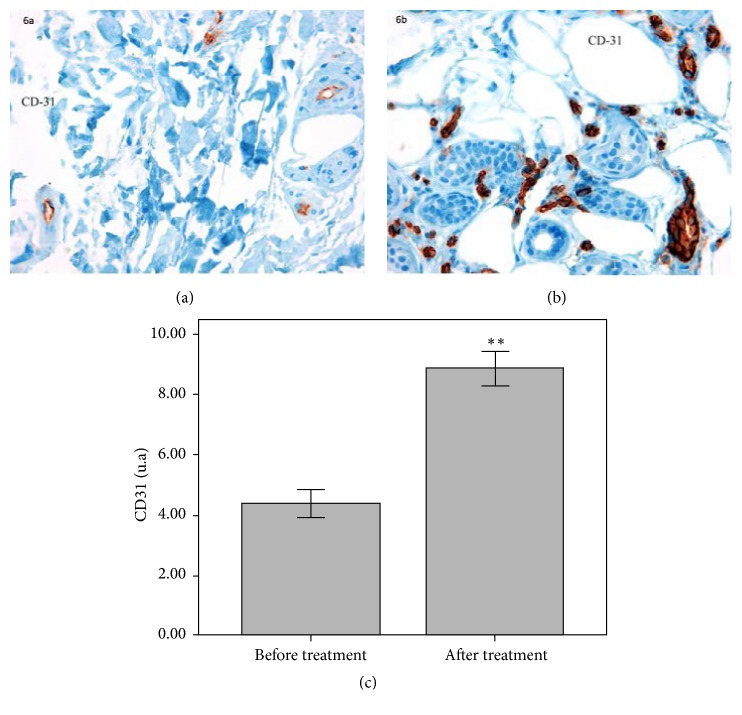
Immunohistochemical staining of the endothelial marker CD-31 before (a) and after treatment (b) showing the increase in vascular presence after cosmetic treatment (40x magnification). Quantification of CD31 positive cells showed a 102.3% increase after treatment compared to baseline (^*∗∗*^*p* ≤ 0.01) (c).

Regarding the analysis of the expression of the endothelial marker Platelet Endothelial Cell Adhesion Molecule 1 (PECAM1 or CD31) (vessels/×40) to address capillarity in the biopsies, a 102.3% increase in the expression of this endothelial marker after treatment (*p* = 0.005) was detected (Figure 6).

**Figure 7:**
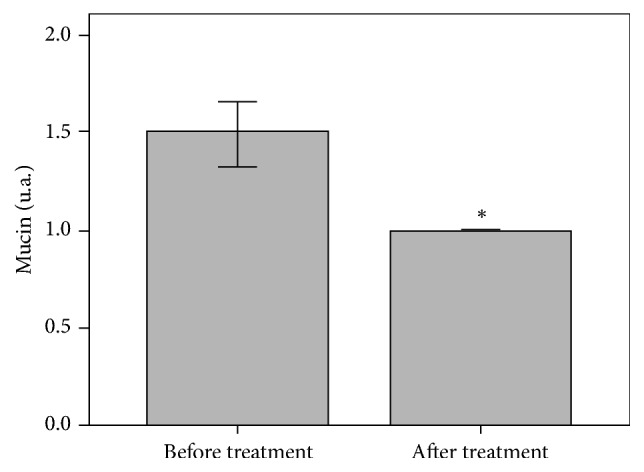
Semiquantitative analysis of mucins showed a 50.0% decrease after treatment compared to baseline (^*∗*^*p* ≤ 0.01).

Finally, a significant decrease of 50% in mucin expression was observed after treatment (*p* = 0.025) compared to baseline (Figure 7).

## 4. Discussion

Photoaging is characterized by epidermal atrophy, which has been linked to ultraviolet-induced damage on the stem cells of the basal layer and the hair follicle [12]. The study product significantly increased epidermal thickness, and this thickening was mediated by the promotion of the epidermal cell proliferation, as it has been demonstrated by the significant increase in the expression of the marker of cell proliferation Ki67 in the basal layer. This stimulus may be attributed either to the retinoid component of the product, as previously described in the literature [13], or to IFC-CAF, which has been reported to facilitate the in vitro proliferation, differentiation, and migration of keratinocytes [9, 14].

Regarding the dermis, collagen type I is the most predominant component of the human dermal ECM [15]. It is synthesized by fibroblasts and is slowly degraded during aging, a process that is accelerated in photoaging [16]. This degradation is mainly mediated by metalloproteinases [17]. During photoaging there is also an accumulation of degenerated elastic fibers combined with other proteins, such as glycosaminoglycans, fibrillin, and lysozyme, which are deposited in the upper dermis triggering elastosis [18]. Knott et al. showed that the activity of fibronectin is reduced after the exposure to ultraviolet and during photoaging [19]. Additionally, the synthesis of collagen is reduced during aging, and it has been postulated that it could be due to fibroblastic senescence [20]. Altogether, these changes promote a decrease in the total number of collagen, elastin, and fibronectin fibers that induces a thinning of the dermis and loss of structural support.

In this study, we did not observe an evident elastosis phenomenon; however, elastin fibers, as well as other ECM components, showed reduced levels as seen by immunohistochemistry. The treatment with the three technologies' combination induced a very significant increase in collagens I and III, elastin, and fibronectin fibers. Moreover, an important increase in the dermal thickness was achieved, although it did not reach statistical significance. Further studies would be necessary in order to assess whether this increase in ECM components is translated into a clinical relevance, such as an increase in elasticity and firmness of the skin.

The stimulus in the synthesis may be due to a direct action of the active ingredients on dermal fibroblasts. According to preliminary studies with WGC, it induces the migration of fibroblasts and increases the expression of collagens III and VII by direct stimulation of fibroblasts [11]. It also increases the expression of TGF-beta, which is known to induce collagen synthesis and inhibit metalloproteinases (data on file). Thus, WGC could be not only increasing the synthesis of ECM components but also avoiding their degradation by inhibiting MMPs, as suggested by the constant levels of the analyzed metalloproteinases (MMP-1 and MMP-3). Finally, WGC induces the expression of hyaluronic synthase (data on file), responsible for the synthesis of hyaluronic acid.

Additionally, IFC-CAF has also been shown to increase the levels of collagen type I and fibronectin [10], so it could be also helping to improve the performance of fibroblasts in secreting ECM components. Moreover, topical retinoids have shown the ability to induce neocollagenesis [21].

Thus, in the present study, the observed increase in the expression of different ECM components is translated into the antiaging clinical effect perceived by both the patients and the investigators.

In photoaged skin, dermal vessels are impaired by the degradation of collagen and elastic fibers, which do not give the necessary support for the correct dermic configuration [22]. In this study, a significant increase in the expression of the endothelial marker CD31 suggests the stimulation of angiogenesis after the treatment. Since this is observed in the absence of inflammation, this angiogenesis would not be secondary to inflammatory process but to skin regeneration, as it is demonstrated by the increase of keratinocyte and fibroblast activity [23].

While dermal mucins provide resistance to the connective tissue under normal conditions, an unusual deposit is observed in some cases, like inflammaging. In our study a significant decrease in the mucin content was demonstrated, which could be related to a decrease in the chronic dermal inflammation associated with photoaging [24].

Regarding the clinical assessments, there is a normal increase of melanin during photoaging, related to an excess of its production by senescent melanocytes. The use of retinoids (and in particular this combination of hydroxypinacolone retinoate with retinol glycospheres) has demonstrated the improvement of hyperpigmentation in previous studies and therefore it may be indicated for the treatment [25]. Even though a clinical improvement in the degree of pigmentation was achieved, an objective assessment to determine its precise scope is necessary. Important changes were obtained regarding hydration, which were in accordance with the perception of both the patients and the investigators.

Tolerance was excellent, which is considered essential for any antiaging product to ensure a proper compliance.

In conclusion, this new cosmetic combination has demonstrated that it is effective for the treatment of skin photoaging, as treatment with it achieved a significant improvement of both histological and clinical parameters. A significant proliferation and epidermal renewal were achieved, as well as replenishment of the components of the dermal extracellular matrix, including collagen, elastin, and fibronectin fibers, which altogether were manifested as a clinical improvement of several signs of photoaging.

## Figures and Tables

**Table 1:** Subjective evaluation of pigmentation and hydration before and after treatment. Pigmentation was more reduced on the treated hand than on the untreated, without reaching statistical significance. Hydration was significantly reduced in the treated hand after treatment compared to baseline (^*∗∗*^*p* ≤ 0.01) and compared to untreated hand after 3 months of moisturization with a standard cream (^++^*p* ≤ 0.01).

	Treated hand	Nontreated hand
Pigmentation before	1.1 ± 0.7	1.3 ± 0.7
Pigmentation after	0.9 ± 0.0	1.2 ± 0.6
Hydration before	2.0 ± 0.7	1.9 ± 0.7
Hydration after	3.0 ± 0.0^*∗∗*^	1.7 ± 0.7^++^
